# Cultural perspectives on children’s tadpole drawings: at the interface between representation and production

**DOI:** 10.3389/fpsyg.2015.00812

**Published:** 2015-06-17

**Authors:** Ariane Gernhardt, Hartmut Rübeling, Heidi Keller

**Affiliations:** Department of Culture and Development, School of Human Sciences, Osnabrück University, Osnabrück, Germany

**Keywords:** tadpole drawings, cultural influence, preschool age, self-drawings, body-proportion-effect, drawing development, artwork

## Abstract

This study investigated tadpole self-drawings from 183 three- to six-year-old children living in seven cultural groups, representing three ecosocial contexts. Based on assumed general production principles, the influence of cultural norms and values upon specific characteristics of the tadpole drawings was examined. The results demonstrated that children from all cultural groups realized the body-proportion effect in the self-drawings, indicating universal production principles. However, children differed in single drawing characteristics, depending on the specific ecosocial context. Children from Western and non-Western urban educated contexts drew themselves rather tall, with many facial features, and preferred smiling facial expressions, while children from rural traditional contexts depicted themselves significantly smaller, with less facial details, and neutral facial expressions.

## Introduction

Tadpole drawings are a pervasive phenomenon of children’s early symbolic development (DeLoache, 2004). They are regarded as the child’s first recognizable drawing of a person, consisting of a round form (head) and two vertical lines attached to it (legs). Tadpole figures emerge around 3 years of age (Cox, 1993) and have been observed in children from Western (e.g., Freeman and Hargreaves, 1977) as well as non-Western countries (Richter, 2001; Rübeling et al., 2011).

Because of the widespread existence of tadpole drawings (Cox, 1993), it is often assumed that they are indicative for general principles regarding children’s graphic development, either expressing deficits in *representation* or deficits in *production* (Cox, 1993). Several authors have argued that tadpole drawings reflect young children’s incomplete representation of the human body (Harris, 1963; Piaget and Inhelder, 1972; Bremner, 1985). However, children have more complete knowledge about body parts than can be inferred from their drawings (Golomb, 1981; Cox, 1993). Hence, problems in the transformation process from mental representations to drawing outcomes were discussed. Freeman (1975, 1980), for example, paid special attention to the positioning of arms in the drawings. In a number of experiments with children from Great Britain, he demonstrated that tadpole drawers, who were asked to add arms to pre-drawn heads and bodies of different sizes, consistently attached them to whatever was larger, head or trunk (Freeman, 1975; Freeman and Hargreaves, 1977; Freeman and Adi-Japha, 2008). Freeman (1975) concluded from the discovery of this so-called *body-proportion effect* that the tadpole figure “is associated with production problems in programming the spatial layout rather than with any peculiar conceptual scheme” (p. 417). In view of the high stability of the body-proportion effect across experimental conditions, it is implicitly taken as a universal phenomenon (e.g., Schoenmackers, 1996; Machón, 2013), although Freeman (1980) himself conceded that his conclusions might not be transferable to all cultures. Indeed, there is no study known to us that has investigated the body-proportion effect in tadpole drawings from various cultures.

However, even if children might construct the tadpole figure following the same general production principles (e.g., body-proportion effect), this does not preclude cultural influences on other aspects of the drawing. In fact, a comparative analysis of tadpole figures drawn by 4–7 years old Madagascan Mahafaly and German children provided first evidence for cultural variation (Liebertz et al., 2001). The authors found that German children drew tadpoles about 44% taller and with more head details than the Madagascan children. These findings are in line with comprehensive cross-cultural research on conventional human figure drawings demonstrating substantial cultural differences with regard to figure size (Meili-Dworetzki, 1981; Aronsson and Andersson, 1996; Payne, 1996; Richter, 2001), as well as variety and shape of details (Wilson and Wilson, 1984; La Voy et al., 2001; Cherney et al., 2006; Yusuf, 2010).

Jolley (2010) outlined three possible sources for these differences: children’s perceptual input (e.g., pictorial models, art, and media), drawing experiences (e.g., availability of drawing material), and learning environments (e.g., instructions, art education, and caregiver-child interactions). In contrast to studies that considered only one of these sources to explain cultural variability (e.g., Aronsson and Andersson, 1996; La Voy et al., 2001; Richter, 2001), the present study proposes a more integrated model. In particular, we apply an ecosocial approach of development (Whiting, 1963; LeVine, 1974; Berry, 1976; Bronfenbrenner, 1989; Keller, 2007), conceptualizing children’s learning environments as ecosocial contexts that vary largely with differences in socioeconomic circumstances (LeVine, 1974; Berry, 1976; Keller, 2007) and experiences of formal schooling (e.g., Greenfield and Childs, 1991; LeVine et al., 1996). These differences are reflected in cultural norms and values, which are organized in comprehensive cultural models (Kağitçibaşi, 2007; Keller, 2007), operating as psychological mindsets for socialization practices. Through participation in these practices, children acquire the respective cultural model in daily interactions from early on (e.g., Rogoff, 2003). The resulting cultural specificity of children’s developmental pathways and emerging concepts of persons has implications for several drawing features of children’s depictions of themselves.

First, with regard to the facial expression of emotions, previous studies have shown that already young children are able to depict different emotional states by varying the shape of the drawn mouth (Buckalew and Bell, 1985). Thus, in line with the cultural variability of emotional expressions and responses (Markus and Kitayama, 1991; Matsumoto, 1991; Tsai et al., 2006; Mesquita, 2007), differences have been observed in children’s depiction of the mouth in more elaborated self-drawings (La Voy et al., 2001; Gernhardt et al., 2013, 2014b). Likewise, variability in the number of depicted facial features across cultures has been explained by differences in children’s early interactional experiences. In particular, smaller number of facial details were related to hierarchical models and the cultural norm of obedience which often demand the avoidance of direct face-to-face interactions (Arnoud, 1981). Finally, with regard to figure size, former studies have shown that the depicted size varies with its perceived importance and emotional valence (Craddick, 1961, 1963; Aronsson and Andersson, 1996). In line with these results it was found that cultural differences in regard to the accentuation of the child’s uniqueness and autonomy versus his interdependence with family members are reflected in the size of self-drawings. Taller figures were found in cultural environments which emphasize independence and uniqueness (Rübeling et al., 2011; Gernhardt et al., 2014b).

Cross-cultural research mainly focuses on three different ecosocial contexts, i.e., Western urban educated families, with high levels of formal education, late parenthood, few children, and a nuclear family constellation, non-Western rural subsistence based farmers, with low levels of formal education, early parenthood, many children, and extended multigenerational households, and non-Western urban educated families, with high levels of formal education and an intermediate age at first birth, number of children, and household composition (e.g., Kağitçibaşi, 2007; Keller, 2007).

In the Western urban educated context, family members are conceptualized as separate individuals with a strong focus on their mental states and personal traits. They strive for economic and emotional independence, which is reflected in the endorsement of traits such as self-confidence, independence, competitiveness, assertiveness, and uniqueness (Markus and Kitayama, 1991). Correspondingly, socialization is primarily child-centered, with caregivers supporting children’s self-initiated activities and positive emotionality as well as responding to the child’s wishes and preferences from early on (Keller and Otto, 2009). Similarly, the official pedagogical orientation in early child-care is characterized by a child-centered, co-constructivist approach (Bredekamp and Copple, 1997). Children are expected to take responsibility for their own learning and teachers are understood as their learning companions (e.g., Fthenakis and Textor, 2000; Schäfer, 2005). Drawing and arts usually describe an integral part of the child-care curricula (e.g., Germany: Billmann-Mahecha, 2014; Sweden: Taguma et al., 2013). Drawing material is freely accessible and children can choose, when and what they draw. Direct instructions, the assignment of topics, and the formulation of drawing rules are rather unusual (Taguma et al., 2013; Rübeling, 2014).

In the non-Western rural traditional context, family members are conceptualized as an inseparable social unit with a strong focus on the fulfillment of existing norms and roles. They are economically and socially interrelated, which is reflected in the endorsement of traits and values such as cooperation, share of resources, and responsibility for the group. Socialization emphasizes children’s adaptation to norms and values and favors apprenticeship-based strategies, such as training, control of emotions, and role modeling (Rogoff, 1990). This didactic approach (Bredekamp and Copple, 1997) is also prevalent in early child-care, characterized by highly structured teaching strategies, which are based on repetition and reinforcement (e.g., rural Cameroon: Gernhardt et al., 2014a). Most commonly, paper and pencils are only rarely available; instead, children often use boards and chalk for drawing and writing (Rübeling, 2014). The teachers usually determine the drawing topic and directly instruct and correct the children.

In the non-Western urban educated context, the Western-oriented education and economic system enhances individuality and psychological autonomy (LeVine et al., 1996), while simultaneously, traditional values, and interrelated patterns within the family are highly valued and maintained (Chaudary, 2004; Kağitçibaşi, 2007). Correspondingly, children are encouraged to be independent and assertive and, at the same time, to be respectful and responsible within the family. Preschool education can be typically described as a mixture of teacher-centered and child-centered approaches, though in urban regions it is an ongoing trend to adjust more and more to Western child-care curricula (e.g., Turkey: Tobin, 2005; Estonia: Tulviste and Kikas, 2010; Costa Rica: UNESCO, 2007). Similar to Western contexts, the development of art education is anchored in the national curricula and drawing material is permanently available.

In the present study we aimed at investigating how the ecosocial context influences preschool children’s tadpole drawings. Based on previous studies that had substantiated the theoretical framework of cultural models across and within national boarders according to the equivalence of ecological and sociodemographic profiles (Keller et al., 2006), we selected tadpole self-drawings from seven cultural groups, located at different continents, each one representing one of the described ecosocial contexts. In particular, we collected children’s drawings from Western educated families living in large cities in Germany and Sweden, non-Western traditional families from rural regions in Cameroon and India, and non-Western educated families from the capitals of Turkey, Costa Rica, and Estonia. The latter classification is based on relevant results showing notable similarities in regard to socialization values and practices of child rearing. In particular, child rearing values and familial education in Estonia has been shown to put greater emphasis on traditional values and conformity compared with Scandinavian orientations. Socialization is described as manifesting the pattern of autonomous relatedness (Tulviste et al., 2007; Tougu et al., 2011; Tulviste, 2013). Comparable results were obtained for urban Turkish mothers (Gernhardt et al., 2013) and urban Costa Rican parents (Keller et al., 2006).

Based on the assumption that the basic structure of tadpole drawings results from general production principles, we do not expect differences between and within ecosocial contexts for the proportion of head-size to the rest of the figure (“head-to-legs ratio”) analogous to the head-to-body ratio used in experimental studies (Freeman, 1980). Furthermore, we do not expect differences between and within ecosocial contexts for the body-proportion effect.

We expect cultural differences for figure size, facial features, and emotional expression. In particular, tadpole self-drawings from children living in Western urban educated contexts are expected to (1) be taller, (2) contain more facial features, and (3) more often include smiling facial expressions as compared to tadpole depictions from non-Western rural contexts. With respect to tadpole self-drawings from children living in non-Western urban educated contexts, mean figure sizes, number of facial details, and the proportion of smiling facial expression are expected to be similar to those from children living in Western urban educated contexts, as has been observed before in conventional human figure drawings (Gernhardt et al., 2013). We further expect that drawing features do not differ significantly between cultural groups that share the same ecosocial context.

## Materials and Methods

### Participants

Drawings were collected from a total of 924 children between 3 and 6 years of age from the three different ecosocial contexts. For the purpose of the present study and in order to ensure representational equivalence, children were included only if their self-drawings met the commonly accepted criteria of a tadpole figure. That is the depiction of a round form (head) and two vertical lines (legs) attached to it without depicting a separate sign for the trunk, while other features like arms, hands, feet, and facial details may be added (Freeman, 1980; Cox, 1993). A total of 183 drawings were classified as tadpole drawings (Western urban educated context: 19.3%; non-Western urban educated context: 27.2%; non-Western rural traditional context: 19.2%). The remaining drawings were part of comparative analyses in previous studies (Rübeling et al., 2011; Schröder et al., 2011; Gernhardt et al., 2014b).

The seven cultural groups did not differ in age, *χ*^2^(6, *N* = 174) = 10.95, *p* = 0.090 (Kruskal-Wallis-Test) and gender distribution, *χ*^2^(6, *N* = 181) = 3.49, *p* = 0.745, with 56.4% male and 43.6% female children in the total sample. However, in line with the postulated differences of ecosocial contexts, Kruskal-Wallis-Tests demonstrated that the cultural groups varied significantly from each other with respect to mothers’ age at first birth, *χ*^2^(6, *N* = 141) = 64.80, *p* < 0.001, mothers’ years of formal education, *χ*^2^(6, *N* = 154) = 79.04, *p* < 0.001, number of siblings, *χ*^2^(6, *N* = 169) = 23.87, *p* = 0.001, number of persons living in the same household, *χ*^2^(6, *N* = 157) = 53.58, *p* < 0.001, and age of mother, *χ*^2^(6, *N* = 148) = 36.18, *p <* 0.001 (see Table 1).

**Table 1:** **Sociodemographic characteristics of the final sample: means and standard deviations**.

	**Western urban educated context**		**Non-Western urban educated context**			**Non-Western rural traditional context**	
	**Urban Germany (*n* = 54)**	**Urban Sweden (*n* = 12)**	**Urban Turkey (*n* = 32)**	**Urban Costa Rica (*n* = 12)**	**Urban Estonia (*n* = 9)**	**Rural Cameroon (*n* = 53)**	**Rural India (*n* = 11)**
Age of child (months)	51.9 (7.9)	48.7 (0.7)	50.6 (7.5)	48.0 (1.0)	49.3 (1.5)	51.4 (5.1)	56.3 (9.5)
Age of mother	35.8 (4.5)	37.0 (4.6)	35.0 (4.4)	30.8 (4.4)	35.5 (7.5)	31.0 (7.9)	28.1 (3.7)
Mothers’ age at first birth	29.9^a^ (4.9)	30.8^a^ (3.1)	30.2^a^ (5.1)	25.6^a^^b^ (4.1)	25.8^a^^b^ (4.1)	21.9^b^ (3.9)	22.7^b^ (2.7)
Mothers’ years of formal schooling	11.7^a^ (1.4)	11.9^a^ (1.4)	11.5^a^ (1.3)	10.1^a^ (1.7)	11.9^a^ (0.4)	7.1^b^ (2.7)	9.7^a^ (3.1)
Number of siblings	0.9^a^ (0.8)	1.2^a^^c^ (0.7)	0.5^a^ (0.5)	0.7^a^ (0.5)	1.3^a^^c^ (1.1)	2.0^b^^c^ (1.9)	1.0^a^^c^ (0.6)
Household size	3.8^a^ (0.9)	4.2^a^ (0.8)	3.8^a^ (0.7)	4.4^a^ (1.4)	4.4^a^ (1.4)	6.4^b^^c^ (2.4)	5.4^a^^c^ (1.5)

Sociodemographic information was not available for the complete samples across measures. Different subscripts indicate significant differences between cultural groups (post hoc comparisons with Bonferroni adjustment).

The Western urban educated context was represented by 54 German children living in Berlin or Osnabrueck and 12 Swedish children from Stockholm. The mothers of these children had high levels of formal education and held a comparable age of about 30 years at first birth; all families but one lived in a nuclear family constellation. Only two German children and one Swedish child had more than two siblings.

The non-Western urban educated context was represented by 32 Turkish children living in Ankara, 12 Costa Rican children living in San José, and nine Estonian children living in Tallinn. The sociodemographic profiles of their mothers differed most from the German and Swedish urban samples with regard to the age at first birth. In particular, mothers from Costa Rica and Estonia were about 4–5 years younger than German and Swedish mothers. Though mean household size is similar to the German and Swedish urban sample, a greater proportion of these families lived in extended households (Ankara: 23.5%; San José: 33.3%; Tallinn: no information available).

The non-Western rural context was represented by 53 rural Nso children living in small villages around Kumbo in the northwestern province of Cameroon and 11 Indian children from a rural region in Western Rajasthan. Most of the Nso mothers (78%) and one third of the Rajasthani mothers attended only elementary school or did not go to school at all. On average, the Nso and Rajasthani mothers were younger at first birth compared to all other samples and more than half of the families lived in extended households (Nso: 54%; Rajasthan: 64%). The number of siblings, however, differed between the Cameroonian and Indian sample. While the Indian children had about the same number of siblings as the other cultural groups, the Cameroonian children had significant more siblings on average. Concurrently, the Indian mothers were younger on average as compared to the Cameroonian mothers (see Table 1), which may be associated with this difference.

### Procedure

Children were recruited in nursery schools, which were randomly contacted by local research assistants. Parents who allowed their child to participate handed back the informed consent to the head of the nursery school and completed a sociodemographic questionnaire. Although no ethic committee surveyed the study, it was carried out in line with the funding agencies ethical principles (German Research Council and Baltic Sea Foundation), which had partly supported the present study. Native research assistants conducted the assessment either at home (Tallinn, Stockholm, and San José) or in nursery school (Berlin, Osnabrueck, rural Nso, rural Rajasthan, and Ankara). The settings were comparable in that the children accomplished the drawing tasks individually in a separate room as soon as they felt at ease with the research assistant. In case of the children from rural Cameroon and India however, who were not used to spend time alone, the procedure was adapted so that children drew in small groups with up to seven children. In order to prevent copying effects, they were seated at a distance from each other and completed the tasks in different orders.

The drawing material consisted of a pencil and white sheets of paper of A4 format (210 mm × 297 mm). Although probably the exposure to drawing materials were different in the cultural groups, it could be assumed that all participants had at least some experiences with paper and pencil. Within all nursery schools, drawing with pencils on paper is used to prepare children’s learning to write. Moreover, even if drawing skills differed due to differences in familiarity with paper and pencils, it would not affect the structural aspects of self-drawings we were analyzing.

The drawing materials were placed vertically in front of the children and they were asked to draw a picture of themselves without time limit. They were instructed in their native language as follows: “Draw yourself, draw a picture of yourself.” Besides, the children had to accomplish four more drawings, one of their family and three copying tasks, which are not part of the present study.

### Coding Procedure

Two independent and trained German research assistants coded all drawings. They were blind to the study hypotheses and to the identity of the drawings. Reliability was computed on 20% of the drawings.

#### Figure Size, Head Size, and Size of Legs

All sizes were measured in millimeters (mm) along an imaginary vertical axis. *Figure size* was defined as the lowest point of the figure to the top line of the head; *head size* as the lowest point of the head to the top line, excluding hairs; *size of legs* as the lowest point of the figure to the top line of the legs. The interclass correlation coefficient (ICC) between the two raters was *r*_ICC_ = 0.99 (figure size), *r*_ICC_ = 0.98 (head size), and *r*_ICC_ = 0.94 (size of legs). Furthermore, a *head-to-legs ratio* was calculated for each child by dividing head size by leg size.

#### Facial Features

*Facial details* included the depiction of eyes, eyebrows, and ears, which were separately coded as 0 (omitted), 1 (one element is present, e.g., one eye), or 2 (both elements are present). Nose, mouth, hair, and teeth were each coded as 0 (omitted) or 1 (present). The sum of all facial details served as final measure. Inter-rater reliability was *r*_ICC_ = 0.96. *Facial expression* was coded only if the mouth was present. A smile was coded if both corners of the mouth turned upward. All other depictions of the mouth were recorded as “no smile.” Inter-rater agreement was *κ* = 0.83.

#### Position of Arms

*Position of arms* was coded only if both arms were present. If the child attached the arms to the legs, it was coded as 1, and if the child attached the arms to the head, it was coded as 2. Inter-rater agreement was *κ* = 0.92

## Results

The statistical analyses are presented with respect to differences on the aggregated level of ecosocial contexts (Western urban educated, non-Western urban educated, and non-Western rural traditional context) as well as on the level of single cultural groups (e.g., German urban and Swedish urban children), which were nested within the ecosocial contexts (e.g., Western urban educated context).

### Body-proportion Effect in Free Drawings

An overview of the descriptive statistics concerning the body-proportion effect is presented in Table 2. First, a two-level nested fixed-random ANOVA was performed on head-to-legs ratio of cultural groups within ecosocial contexts. No significant differences were found between ecosocial contexts, *F*(2, 176) = 0.52, *p* = 0.633, nor between cultural groups within each ecosocial context, *F*(4, 176) = 0.43, *p* = 0.785, indicating that on average, the children depicted their heads in similar proportion to their legs across cultural groups and ecosocial contexts.

**Table 2:** **Descriptive statistics of the body-proportion effect in free drawings by cultural group**.

	**Western urban educated context**		**Non-Western urban educated context**			**Non-Western rural traditional context**	
	**Urban Germany (*n* = 54)**	**Urban Sweden (*n* = 12)**	**Urban Turkey (*n* = 32)**	**Urban Costa Rica (*n* = 12)**	**Urban Estonia (*n* = 9)**	**Rural Cameroon (*n* = 53)**	**Rural India (*n* = 11)**
Head-to-legs ratio: *M (SD)*	0.80 (0.7)	0.75 (0.4)	0.74 (0.6)	0.81 (0.8)	0.78 (0.7)	0.72 (0.5)	0.65 (0.5)
Pos. of arms (%)^1^:	(*n* = 27)	(*n* = 6)	(*n* = 21)	(*n* = 6)	(*n* = 6)	(*n* = 20)	(*n* = 6)
Head	55.6	66.6	28.6	33.3	50.0	45.0	33.3
Legs	44.4	33.3	71.4	66.6	50.0	55.0	66.6
Body-proportion-effect^2^: r_*pb*_	0.45	0.43	0.57	0.97	0.83	0.51	0.26

1 Statistical analyses within ecosocial contexts were conducted with Fisher’s exact test.

2 Due to small sample sizes, the correlation is only reported on a descriptive level.

Next, among all children who depicted arms, a Pearson chi-square test was conducted to compare the position of arms on either the head or the legs between ecosocial contexts. The analysis demonstrated no significant differences, *χ*^2^(2, *N* = 92) = 4.00, *p* = 0.135. Likewise, further analyses with Fisher’s exact test within ecosocial contexts demonstrated no significant differences between the cultural groups within each ecosocial context (see Table 2). Thus, among those children who depicted arms, a similar proportion of children across ecosocial contexts and cultural groups depicted their arms on the head.

**FIGURE 1:**
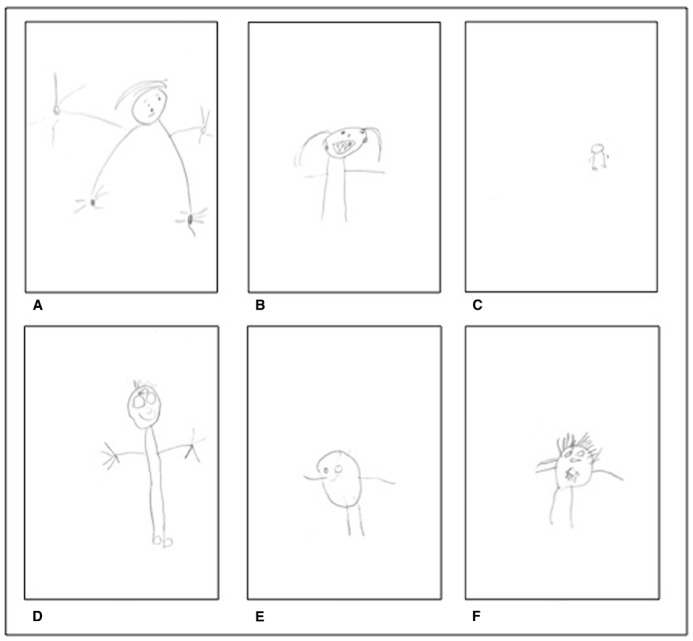
**Examples of tadpole drawings across cultural groups. (A)** Germany urban, boy, age: 4;0. **(B)** Turkey urban, girl, age: 4;1. **(C)** Cameroon rural, boy, age: 4;2. **(D)** Costa Rica urban, boy, age: 4;0. **(E)** Estland urban, girl, age: 4;1. **(F)** India rural, girl, age: 3;11.

Finally, separate point-biserial correlation coefficients (one-tailed) revealed significant positive correlations between positioning arms on the head and the ratio scores within each ecosocial context (Western urban educated: *r*_pb_ = 0.41, *p* = 0.009; non-Western urban educated: *r*_pb_ = 0.66, *p* < 0.001; non-Western rural traditional: *r*_pb_ = 0.47, *p* < 0.008) and within cultural groups (see Table 2). Thus, the larger the head was drawn in comparison to the legs, the more often children positioned arms on the head (see also Figure 1).

### Figure Size and Head Size

Two two-level nested fixed-random ANOVAs were performed respectively on figure size and head size between and within ecosocial contexts. Since the number of facial details may be confounded with head size, the latter was controlled for in the statistical analysis. The analyses revealed significant differences between ecosocial contexts for figure size, *F*(2, 176) = 8.54, *p* = 0.037, ${\text{η}}_{p}^{2}$ = 0.18, and for head size, *F*(2, 176) = 16.76, *p* = 0.014, ${\text{η}}_{p}^{2}$ = 0.10, but not for cultural groups within ecosocial contexts, *F*(4, 176) = 1.49, *p* = 0.21 (figure size) and *F*(4, 176) = 0.43, *p* = 0.79 (head size). *Post hoc* Tukey tests (*p* < 0.05) showed that on average, figure sizes as well as head sizes were significantly higher for children from Western and non-Western urban educated contexts as compared to those from non-Western rural traditional contexts (see Table 3 and Figure 1).

**Table 3:** **Means and standard deviations of figure size and head size (in mm) by cultural group**.

	**Western urban educated context**		**Non-Western urban educated context**			**Non-Western rural traditional context**	
	**Urban Germany (*n* = 54)**	**Urban Sweden (*n* = 12)**	**Urban Turkey (*n* = 32)**	**Urban Costa Rica (*n* = 12)**	**Urban Estonia (*n* = 9)**	**Rural Cameroon (*n* = 53)**	**Rural India (*n* = 11)**
Figure size	143.4 (66.1)	122.4 (48.1)	115.8 (50.0)	146.3 (57.5)	151.4 (46.7)	81.8 (50.0)	77.6 (42.6)
Head size (in mm)	58.5 (42.1)	50.0 (26.6)	49.0 (38.8)	54.2 (37.6)	55.9 (37.2)	32.4 (27.6)	22.4 (15.5)

Because age and gender revealed to be important variables for children’s drawings in previous research, an additional ANCOVA was computed with ecosocial context as independent variable and age and gender as covariates. The analysis demonstrated a significant difference between ecosocial contexts irrespective of children’s age and gender, *F*(2, 173) = 19.82, *p* < 0.001, ${\text{η}}_{p}^{2}$ = 0.19. Further, there was neither a significant effect of age (*p* = 0.13) nor of gender (*p* = 0.72).

### Facial Details

In order to account for the possible impact of head size upon the number of depicted facial details, a one-way analysis of covariance (ANCOVA) was conducted to compare the number of facial details between ecosocial contexts, with head size as covariate. The analysis demonstrated a significant difference, *F*(2, 179) = 4.33 *p* = 0.015, ${\text{η}}_{p}^{2}$ = 0.05, irrespective of head size. *Post hoc* comparisons (*p* < 0.05) yielded that the children from Western and non-Western urban educated contexts drew significantly more facial details than did children from the non-Western rural traditional context (see Table 4). Furthermore, two *t*-tests and a univariate ANOVA were performed to compare the cultural groups within each ecosocial context. However, the analyses showed no significant differences (all *p*’s > 0.05). To account for gender and age effects, an additional ANCOVA was computed with ecosocial context as independent variable and age and gender as covariates. The analysis still revealed significant differences between ecosocial contexts, *F*(2, 173) = 6.27, *p* = 0.002, ${\text{η}}_{p}^{2}$ = 0.07. Further, a significant effect of age was obtained, *F*(1,173) = 12.19, *p* = 0.001, ${\text{η}}_{p}^{2}$ = 0.07.

**Table 4:** **Descriptive statistics of facial details and facial expression by cultural group**.

	**Western urban educated context**		**Non-Western urban educated context**			**Non-Western rural traditional context**	
	**Urban Germany (*n* = 54)**	**Urban Sweden (*n* = 12)**	**Urban Turkey (*n* = 32)**	**Urban Costa Rica (*n* = 12)**	**Urban Estonia (*n* = 9)**	**Rural Cameroon (*n* = 53)**	**Rural India (*n* = 11)**
Facial details: *M* (SD)	3.6 (2.2)	3.5 (1.2)	4.0 (1.9)	3.2 (1.5)	3.7 (1.9)	2.6 (2.1)	3.8 (2.1)
Facial details (%):							
Eyes	87.0	100.0	96.9	83.3	88.9	67.9	90.9
Mouth	55.6	41.7	71.9	66.7	77.8	41.5	45.5
Nose	44.4	41.7	37.5	58.3	44.4	13.2	54.5
Ears	20.4	8.3	15.6	0.0	11.1	26.4	9.1
Hair	35.2	33.3	50.0	8.3	44.4	5.7	27.3
Smiling (%):	56.7	80.0	54.2	75.0	57.1	4.3	20.0

The closer inspection of single facial details with chi-square analyses revealed significant differences concerning the depiction of eyes, *χ*^2^(2, *N* = 183) = 11.33, *p* = 0.003, mouth, *χ*^2^(2, *N* = 183) = 10.30, *p* = 0.006, nose, *χ*^2^(2, *N* = 183) = 9.94, *p* = 0.007, and hair, *χ*^2^(2, *N* = 183) = 16.30, *p* < 0.001. In particular, children from non-Western rural traditional contexts more often omitted eyes (z = 2.5), nose (z = 2.0), and hair (z = 2.7) as compared to the Western and non-Western urban educated context, while the latter more often depicted a mouth (z = 1.8) as compared to the other ecosocial contexts.

Furthermore, separate Fisher’s exact tests were conducted to compare cultural groups within each ecosocial context with regard to single facial details. Significant differences were demonstrated only for nose within the non-Western rural traditional context, *p* = 0.006, and for hair within the non-Western urban educated context, *p* = 0.040. While the Indian children more often included a nose as compared to the Cameroonian children, the Costa Rican children less often depicted hair as compared to the Turkish and Estonian children (see Table 4).

### Facial Expression

A chi-square analysis revealed significant differences in the facial expression between ecosocial contexts, *χ*^2^(2, *N* = 102) = 22.46, *p* < 0.001. Of those children who depicted a mouth, more than half of the children living in Western and non-Western urban educated contexts drew themselves smiling, while only few of the rural children did so (z = –3.0; see Table 4). Moreover, within ecosocial contexts, Fisher’s exact tests revealed no significant differences between the cultural groups within ecosocial contexts.

## Discussion

In view of the high prevalence of tadpole drawings across cultures (Cox, 1993) and the assumed generality of production principles underlying its basic structure (Freeman, 1980), the present study investigated the specific influence of ecosocial contexts upon single features of the drawings. Indeed, the examination of tadpole drawings from seven cultural groups representing three ecosocial contexts indicated both, the existence of general drawing principles, as well as cultural influences upon this early representational form.

In line with the expectation of general production principles underlying the basic structure of the tadpole figure (Freeman, 1980), our data did not reveal significant differences in mean size ratio of head to legs between and within ecosocial contexts, even with regard to the comparably small figures drawn by the Cameroonian and Indian rural children. The findings therefore strengthen the view that the basic vertical structure of the tadpole figure is not affected by the child’s cultural background but rather embodies a universal aspect of children’s mental representation of persons (e.g., Freeman, 1980). Moreover, corresponding to the body-proportion effect, children across and within ecosocial contexts attached arms the more frequently to the head, the taller they drew the head compared to the legs. For the understanding of the tadpole figure as an important step in children’s cognitive development, this result is of double relevance. First, it reveals for the first time that children follow the same drawing rules, irrespective of cultural background. Second, it demonstrates that the body-proportion effect, as it was previously observed for pre-drawn depictions of head and trunk (Freeman, 1980), is also apparent in free tadpole drawings. Thus, the present results confirmed the assumption that young tadpole drawers from different cultural backgrounds apply similar production principles.

However, besides these cross-cultural similarities concerning the basic structure of the tadpole figure, single features of the drawings varied with the ecosocial context. Thereby, cultural differences in figure size, number of facial details, and facial expression not only substantiate the results of previous cross-cultural studies with conventional human figure drawings, it also extends the findings to the earliest form of human figure drawings. The sources of these cultural variations are doubtlessly multifaceted and difficult to disentangle. However, the theory of cultural models (Keller, 2007) provides an integrative and well-confirmed theoretical framework (e.g., Keller and Kärtner, 2013) to understand the observed cultural variation.

Specifically, the present results confirm former findings regarding the importance of cultural norms and values on figure size (e.g., Aronsson and Andersson, 1996; Richter, 2001; Rübeling et al., 2011). From the perspective of cultural models, in Western urban educated contexts, children’s concept of the self is primarily shaped by a child-centered learning environment emphasizing psychological autonomy and independence (Keller, 2007). In line with this view children depicted themselves as comparably tall figures claiming a large part of the paper sheet as their “personal space.” Non-Western rural traditional children, in contrast, learn to view single persons as members of larger hierarchically organized social systems rather than as unique and independent individuals. Their considerably smaller self-depictions are indicative for this view inasmuch as only a small part of the sheet is filled out by the figure. The mean figure size of children from non-Western urban educated contexts was about the same height as from children living in Western urban educated contexts, which may be indicative for the adoption of cultural norms and values, boosted by the steady increase of formal education and economic independence (Kağitçibaşi, 2007). Though this interpretation stresses children’s culturally shaped internal representations of the self (and others), it does not rule out external influences (e.g., instructions, modeling), which may also be indicative for particular cultural models and conveyed in direct caregiver-child interaction.

Although we controlled for age and gender differences and focused on children with the same developmental level of human figure drawing, alternative explanations for this finding should be considered. Specifically, there might be a general tendency of non-Western rural traditional children to draw rather small, due to the lack of experience with paper and pencil or their restricted availability. Nevertheless, previous studies demonstrated that figure size differences between children from non-Western rural and Western urban educated contexts persisted even when controlling for the size of the drawing of a geometric figure (Rübeling et al., 2011) or a non-human object (Gernhardt et al., 2014b). Further, comparably small figure sizes were also obtained from unschooled rural Mahafaly children living in Madagascar who drew on A3 paper sheets (Liebertz et al., 2001).

With respect to the number of facial details and facial expression, the study expectations could be confirmed as well. Tadpole drawers from Western and non-Western urban educated contexts depicted significantly more facial details than did children from non-Western rural traditional contexts, irrespective of the head size. Even though the present effect is only of medium size, the result is consistent with a former study about facial details of conventional drawers in these ecosocial contexts (Gernhardt et al., 2013). Besides, children’s age revealed to be another factor influencing the depiction of facial details, though this effect is also of medium size. This result is in line with findings related to conventional human figure drawings, showing that older children generally draw more (facial) details than younger children (e.g., Cox, 1993). This may be indicative for children’s increasing attention and memory capacity.

Moreover, of those children who drew a mouth, children from Western and non-Western urban educated contexts more often depicted themselves smiling as compared to the non-Western rural tadpole drawers, in line with the instantiation and maintenance of positive emotionality in the former context (Keller and Otto, 2009). It can therefore be concluded that the importance of the face and the respective endorsement of emotional control versus positive emotionality of each ecosocial context already seem to become manifested in young children’s earliest recognizable drawings of themselves. As an alternative explanation, the smaller number of facial details in non-Western rural children’s drawings could be attributed to lower graphical abilities compared to Western urban children. However, even in children’s conventional human figure drawings with more elaborated body details, rural non-Western children often omitted particular facial features (Gernhardt et al., 2013).

Finally, the results of the present study demonstrated that the examined single drawing features varied consistently across ecosocial contexts but not between the cultural groups that belonged to the same ecosocial context. This finding contributes to our understanding of the validity of aggregating cultural groups from different parts of the world to broader ecosocial contexts.

From a more general perspective the results of the present study demonstrate the cultural shaping of young children’s symbolic activity. The starting point is given by children’s universal experience of postural and locomotor activities, demanding a permanent battle with gravitational force (Bronstein, 2009). One essential outcome of this experience is the child’s implicit knowledge of the vertical structure of the human body, which seems to be reflected in the top down arrangement of head and legs in the tadpole figure (Freeman, 1980). Another outcome is that children perceive legs (and arms) as mobile extensions from a solid entity. In the tadpole figure, this knowledge is reflected in separate lines attached to a rounded form. So far, the tadpole figure conveys a basic graphic scheme, which is shared by healthy children living in very different cultural environments. However, when children are asked to portray themselves (or a known person) the mere production of this basic scheme is not sufficient, as the task requires the depiction of a real person (e.g., the self), not only a human body. At this point cultural concepts of self and others come into play, elaborating the child’s basic representation of the human being by implementing particular features. This process may be mediated in at least two ways: on the one hand *implicitly* through the child’s emerging, culture-specific concept of persons that result from immediate socialization experiences and on the other hand *explicitly*, for example through instruction, training, and drawing rules. Hence, young children’s symbolic activity as it is demonstrated in early tadpole drawings reveals as a finely-tuned cultural shaping, similar to what has been demonstrated in various other fields of cognition (Nisbett and Norenzayan, 2002).

The study has some limitations. First, the body-proportion effect has not been studied as in the Freeman design. Thereby, the possibility of confounding variables must be considered. For instance, some children may have added arms to the head before completing the whole figure, which would exclude the possibility to attach arms depending on the proportion of head size to leg size. Even though this sequence is an exception in free human figure drawings (Freeman, 1980), the results could be further validated by using the original test conditions in tadpole drawers cross-culturally. Second, although the inclusion of diverse cultural groups is a major strength of this study, the sample sizes are unequal, due to different availability rates of children in the tadpole stage across the cultural groups. Finally, future research should contribute to an in-depth understanding of the social and psychological processes by which cultural norms and values are transformed into children’s human figure drawings. For this purpose, the embedding of children’s drawing activities in social interactions with caregivers, teachers, and peers deserve closer inspection.

Overall, two conclusions can be drawn from the findings of the present study. On the one hand, tadpole drawings seem to underlie some universal production principles. Specifically, the present study substantiates Freeman’s (1980) conclusion that “the human figure, above all, is a design problem” (p. 338). On the other hand, the designing of single features of the tadpole figure is susceptible to cultural influences and may be linked to differences in children’s culturally shaped learning environments. With this, our study contributes to the understanding of cultural similarities and differences as two sides of the same medal.

### Conflict of Interest Statement

The authors declare that the research was conducted in the absence of any commercial or financial relationships that could be construed as a potential conflict of interest.
